# A Novel Method for Amyloid Detection in Human Tissue Load Using a Fluorescent Dye — Congo Red Analogue

**DOI:** 10.17691/stm2020.12.1.08

**Published:** 2020

**Authors:** V.V. Guselnikova, O.I. Antimonova, E.A. Fedorova, M.M. Shavlovsky, A.E. Safray, A.A. Rukavishnikova, V.V. Ilyin, B.L. Milman, D.E. Korzhevskii

**Affiliations:** Senior Researcher, Laboratory of Functional Morphology of the Central and Peripheral Nervous System, Department of General and Special Morphology, Institute of Experimental Medicine, 12 Akademika Pavlova St., Saint Petersburg, 197376, Russia; Junior Researcher, Laboratory of Human Molecular Genetics, Department of Molecular Genetics, Institute of Experimental Medicine, 12 Akademika Pavlova St., Saint Petersburg, 197376, Russia; Researcher, Laboratory of Functional Morphology of the Central and Peripheral Nervous System, Department of General and Special Morphology, Institute of Experimental Medicine, 12 Akademika Pavlova St., Saint Petersburg, 197376, Russia; Professor, Head of the Laboratory of Human Molecular Genetics, Department of Molecular Genetics, Institute of Experimental Medicine, 12 Akademika Pavlova St., Saint Petersburg, 197376, Russia; Leading Researcher, Cardiomyopathy Laboratory, Institute of Cardiovascular Disease, Pavlov University, 6-8 L’va Tolstogo St., Saint Petersburg, 197022, Russia; Professor, Department of Medical Genetics, North-Western State Medical University named after I.I. Mechnikov, 41 Kirochnaya St., Saint Petersburg, 191015, Russia; Head of the Forensic Histology Department, The Leningrad Region Bureau of Forensic Medical Expertise, 36-38-40 “B” Shkapina St., 198095, Saint Petersburg, Russia; Lecturer, Department of Forensic Medicine and Law Science, Pavlov University, 6-8 L’va Tolstogo St., Saint Petersburg, 197022, Russia; Expert, Forensic Histology Department, The Leningrad Region Bureau of Forensic Medical Expertise, 36-38-40 “B” Shkapina St., 198095, Saint Petersburg, Russia; Lecturer, Department of Forensic Medicine and Law Science, Pavlov University, 6-8 L’va Tolstogo St., Saint Petersburg, 197022, Russia; Researcher, Laboratory of Synthesis and Nanotechnology of Drugs, S.V. Anichkov Department of Neuropharmacology, Institute of Experimental Medicine, 12 Akademika Pavlova St., Saint Petersburg, 197376, Russia; Head of the Laboratory of Biomedical and Pharmaceutical Mass-Spectrometry, Institute of Experimental Medicine, 12 Akademika Pavlova St., Saint Petersburg, 197376, Russia; Professor of Russian Academy of Sciences, Head of the Laboratory of Functional Morphology of the Central and Peripheral Nervous System, Institute of Experimental Medicine, 12 Akademika Pavlova St., Saint Petersburg, 197376, Russia; Leading Researcher, Cardiomyopathy Laboratory, Institute of Cardiovascular Disease, Pavlov University, 6-8 L’va Tolstogo St., Saint Petersburg, 197022, Russia

**Keywords:** amyloid, disodium salt of 2,7-(1-amino-4-sulfo-2-naphthylazo)fluorene, Congo red, fluorescence microscopy, amyloidosis, human myocardium

## Abstract

**The aim of the study** was to develop a new technology for the detection of amyloid in human tissues based on the fluorescent dye, disodium salt of 2,7-(1-amino-4-sulfo-2-naphthylazo)fluorene (DSNAF).

**Materials and Methods:**

Synthesis of DSNAF was performed by diazotization of 2,7-diaminofluorene in a stream of argon followed by azo coupling with naphthionic acid. Identification of DSNAF was performed using MALDI mass spectrometry. Human myocardial samples from males and females aged from 85 to 98 years (n=11) were the material for the histochemical study. Myocardial paraffin sections were stained with a 0.1% aqueous solution of Congo red or with an aqueous solution (0.1 or 0.034%) of DSNAF under the same conditions.

**Results:**

It has been demonstrated for the first time that a new fluorene-based analogue of Congo red, DSNAF, can be successfully used to identify amyloid deposits in histological sections of human myocardium. In terms of the specificity and intensity of amyloid staining, DSNAF is comparable to Congo red, which is the gold standard for detecting amyloid deposits. The fluorescence intensity of DSNAF when binding to amyloid fibrils is significantly higher than the intensity of Congo red fluorescence (with a lower intensity of background fluorescence of heart muscle tissue). This is especially useful for identifying small deposits of amyloid in the human tissues which is important when using small biopsies.

**Conclusion:**

The advantages of using DSNAF allow us to consider the developed technology for the detection of amyloid as a new promising method of identifying amyloid deposits in human tissues.

## Introduction

Amyloidosis is a group of conformation diseases the common sign of which is the deposition of a special substance called amyloid in organs and tissues [1]. Amyloid is a complex formation. Its main component is aggregated proteins in the form of fibrils. Presently, 36 human proteins forming amyloid and, respectively, 36 types of amyloidosis are known [2]. Some types of amyloidosis are accompanied by heart injury, amyloid cardiomyopathy [3–6]. In this case, interstitial amyloid deposits are found in the myocardium of atria and ventricles of the heart, sinoatrial and atrioventricular nodes, His bundle branches, middle and outer coat of intramural coronary arteries and veins [7, 8]. Absence of strictly specific symptoms makes the diagnosis of cardiac amyloidosis difficult, therefore, if amyloid cardiomyopathy is suspected according to the findings of the noninvasive methods, it is recommended to perform endocardial biopsy with subsequent histochemical analysis of the samples [4, 8]. Staining with Congo red and thioflavin T are the widely used histochemical methods of amyloid identification [9–11]. However, these methods have some disadvantages which complicate verification of amyloid aggregations in bioptates and sectional material [12]. In this connection, search for new dyes for effective amyloid identification remains a vital task.

One of the approaches to improve amyloid detection is to apply analogues of Congo red and thioflavin T [13]. It has been shown by us that one of the fluorene-based analogues of Congo red, disodium salt of 2,7-(1-amino-4sulfo-2naphthylazo)fluorine (DSNAF) can be successfully applied to identify amyloid deposits in the tissues of laboratory animals [14–16]. The possibility of using this stain to detect amyloid in the human tissues remains unclear.

**The aim of the study** was to develop a new technology for the detection of amyloid in human tissues based on the fluorescent dye, disodium salt of 2,7-(1-amino-4-sulfo-2-naphthylazo)fluorene (DSNAF).

## Materials and Methods

***Synthesis and identification of DSNAF.*** Synthesis of DSNAF was performed by diazotization of 2,7-diaminofluorene (1 molar equivalent) in a stream of argon followed by azo coupling with naphthionic acid (2 molar equivalents). The reaction product was precipitated in the acidic medium by sodium chloride. Purification was performed on a high performance liquid chromatography system BioLogic Duo Flow (Bio-Rad Laboratories, Inc., USA) using a reversed phase column in acetonitrile gradient. The product was subject to lyophilization.

DSNAF was identified with the help of MALDI mass spectrometer UltrafleXtreme MALDI TOF/TOF (Bruker Daltonics, Germany) using HCCA matrix. Spectra were obtained under the action of ultraviolet laser with 1000 Hz frequency and 30–40% power, 1500–2500 laser pulses, in the reflective mode, in the m/z range (relation of ion mass to its charge) from 400 to 1000 Da. The measurement accuracy of ionic masses in the range of m/z 650–750 Da (the region of protonated and cationized molecules) was not worse than 3–5 ppm.

**Figure 1:**
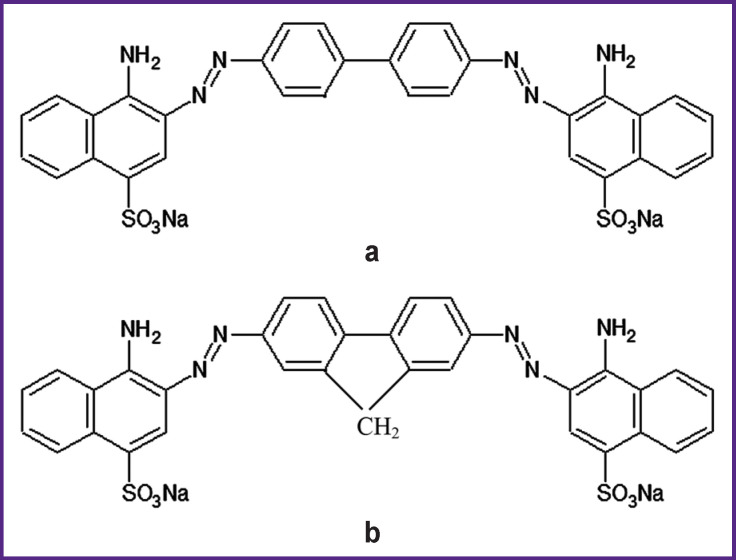
The molecule structure of Congo red (a) and DSNAF 2,7-(1-amino-4-sulfo-2-naphthylazo)fluorine (b)

Structural formulas of Congo red and DSNAF molecules are presented in Figure 1.

***Histochemical investigation.*** Histochemical investigations were conducted using myocardial samples from men and women (n=11) aged 85–98 years taken from the archives of the Laboratory of Functional Morphology of the Central and Peripheral Nervous System of the Institute of Experimental Medicine and Forensic Histology Department of the Leningrad Region Bureau of Forensic Medical Expertise. The investigation was approved by the Ethical Committee of the Institute of Experimental Medicine. The samples were fixed in 10% buffered formalin which is used as a standard for pathoanatomical examinations [17] and embedded in paraffin according to the standard technique. The paraffin blocks were cut into 5 μm-thick sections. The sections were dewaxed in xylenes and rehydrated in alcohols in descending order of concentration, and after the application of Ehrlich’s hematoxylin solution, they were incubated at room temperature for 1 min. Once the incubation was completed, the dye was removed from the sections, the preparations were rinsed in distilled water, then in ammonia water (in order “to blue” hematoxylin), and washed in distilled water for 5 min at constant stirring. Thereafter, the required amount of the dye (0.1% Congo red aqueous solution (Sigma-Aldrich, USA) or 0.1% or 0.034% DSNAF aqueous solution) was applied on the sections, which were placed into the moist chambers, and incubated for 30 min at 27°С. The dye then was removed from the sections and the preparations were washed in 2 changes of distilled water (for 10 min each). After washing, the sections were put into the water soluble medium Ultramount or Fluorescence Mounting Medium (Dako, Denmark).

The method of staining paraffin tissue sections with DSNAF solution to visualize amyloid using fluorescent microscopy has been patented [18].

## Results

**Figure 2:**
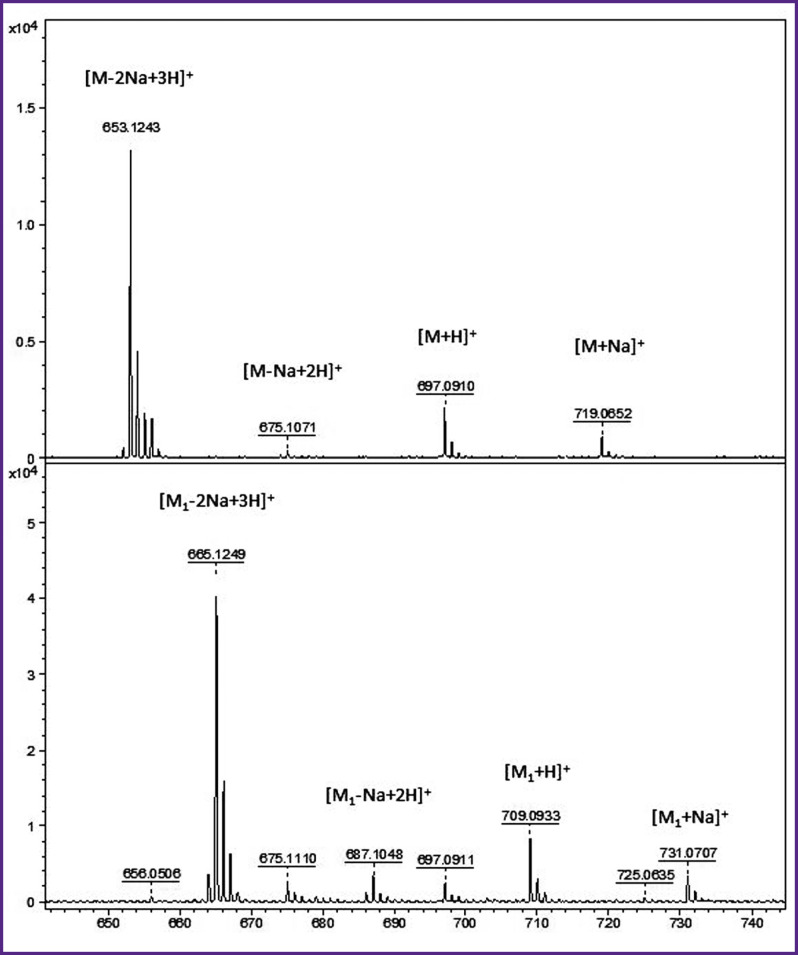
Mass spectra (the region of protonated and cationized molecules) of Congo red (*upper spectrum*) and DSNAF (*lower spectrum*) X-axis: relation of mass to ion charge (m/z, Da), Y-axis: peak intensity (standard unit). The composition of the main ions is indicated

***Results of mass spectroscopic analysis.*** The molecule structure was verified using MALDI mass spectrometry. In case of Congo red, the mass spectrum contained four signals corresponding to different ionic forms of this substance (Figure 2, *upper spectrum*). The precise measurements of ionic masses agreed with the molecular formula of Congo red: С_32_H_22_N_6_Na_2_O_6_S_2_ (М). In case of DSNAF, four similar peaks were present in the spectrum (Figure 2, *lower spectrum*) the mass numbers of which were 12 Da greater. Precise measurements of ionic masses confirmed the DSNAF molecular formula: С_33_H_22_N_6_Na_2_O_6_S_2_ (М_1_) which contained one carbon atom more than the molecule of Congo red. The main signals of Congo red were not evident or low signifying absence of Congo red admixture in the DSNAF solution.

**Figure 3:**
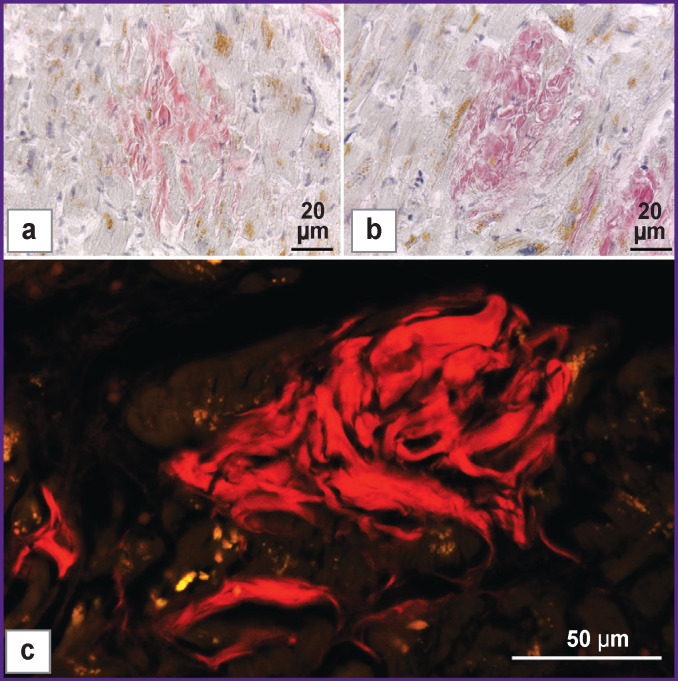
Amyloid aggregations in the human myocardium: (a) staining with 0.1% solution of Congo red, nuclear counterstaining with Ehrlich’s hematoxylin; (b), (c) staining with 0.1% solution of DNSAF, nuclear counterstaining with Ehrlich’s hematoxylin; (a), (b) microscopy in the transmitted light (×10); (c) fluorescence microscopy (×40). *Red color* — fluorescence of amyloid deposits stained with DSNAF; *yellow color* — autofluorescence of lipofuscin in cardiomyocytes

***Results of histochemical analysis.*** Analyzing the Congo red-stained preparations in the transmitted light it has been found that in patients with diagnosed amyloidosis of the heart, aggregates of amyloid in the myocardium were found in the interstitium between the muscular fibers, intermuscular layers of the connective tissue, and in the blood vessel walls. When Congo red was used as a dye (0.1% solution), these aggregates became pink-colored (Figure 3 (a)). The cardiac muscular tissue acquired a bluish grey color.

When DSNAF-stained preparations were analyzed in the transmitted light, this dye has been shown to effectively identify amyloid in the human myocardium as well (Figure 3 (b)), its aggregates being well identified already at a low microscope magnification (×10). If DSNAF was used, amyloid acquired a deeper reddish (crimson) color. Intensity of staining amyloid with DSNAF is comparable with that of staining with Congo red. The cardiac muscular tissue had bluish grey color similar to the use of Congo red (see Figure 3 (b)). In the course of the comparative analysis of the series sections, no differences in the amount and distribution of amyloid aggregations were found irrespective of using DSNAF or Congo red dye.

Fluorescent microscopy showed that DSNAF fluoresced in the red spectrum range when binding to amyloid fibrils in the human myocardium (Figure 3 (c)). And only low background fluorescence of the muscular tissue was observed which did not hinder the identification of amyloid aggregations even at a low magnification (×10). Significant reduction of the background fluorescence of the muscular tissue occurred at low DSNAF concentrations (0.034% solution). At the same time, a high level of amyloid fluorescence was preserved. It simplified the identification of amyloid aggregations in the myocardium when fluorescence microscopy was employed.

**Figure 4:**
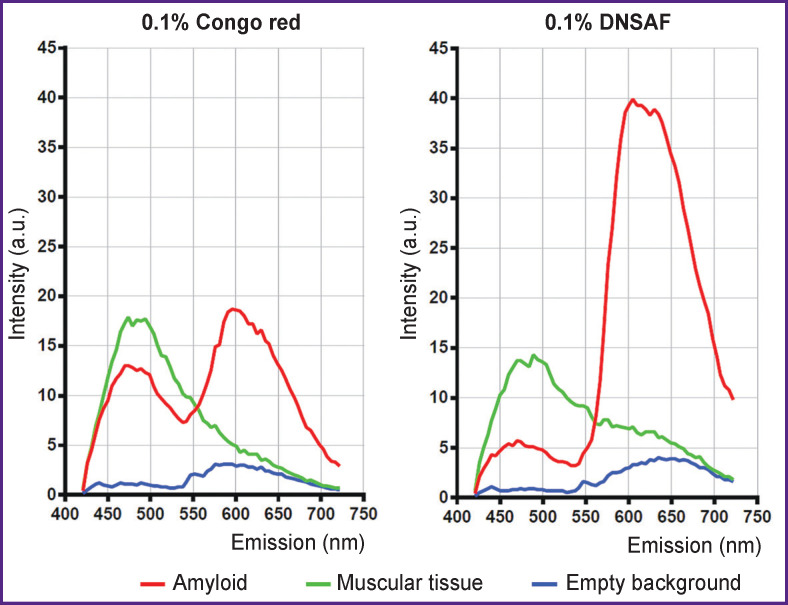
Spectral characteristics of Congo red and DNSAF The diagrams show fluorescence intensity of the dye (Congo red or DSNAF) in case of binding to amyloid fibrils (*red lines*), intensity of background fluorescence of the muscular tissue (*green lines*), and intensity of background fluorescence on the preparation regions free of the tissue (fluorescence of the embedding media, *blue lines*). The diagrams were built based on the quantitative data of fluorescence intensity obtained by means of confocal laser microscope and ZEN2011 microscope software (Zeiss, Germany) at identical microscope settings

Differences in fluorescence intensity of Congo red and DSNAF have been established using confocal laser microscopy. Quantitative values of fluorescence intensity of Congo red and DSNAF bound to amyloid were obtained (Figure 4, *red lines*) as well as the values of background fluorescence of the cardiac muscular tissue (Figure 4, *green lines*). Fluorescence measurements in those places where there was no tissue on the preparations (fluorescence of the embedding medium) served as the control (Figure 4, *blue lines*).

Maximal fluorescence intensity of amyloid aggregations stained with DSNAF has been shown to be more than twice higher than that observed with the use of Congo red (see Figure 4, *red lines*). But unlike Congo red which, when applied, showed two peaks of fluorescence (460 and 600 nm), DSNAF has only one fluorescence peak characterized by the presence of a small plateau (600–640 nm). The intensity of the background fluorescence of the muscular tissue after staining with DSNAF was lower relative to the case of Congo red application (see Figure 4, *green lines*). Background fluorescence in the places of the preparation containing no tissue was characterized by low values of intensity growing slightly in the region of 550–650 nm when stained with Congo red and in the region of 550–700 nm for the DSNAF dye (see Figure 4, *blue lines*).

## Discussion

At present, staining with Congo red is considered the gold standard for amyloid identification [2, 19]. The amyloid diagnosis is based on the detection of congophilic deposits which are visualized as apple green fluorescent structures against a dark background on the samples viewed in polarized light [1, 12]. But the ability of Congo red to fluoresce when binding to amyloid fibrils noted by Cohen et al. as long ago as 1959 [20] is not used in the practice of diagnostic investigations. However, this property can be employed to detect small aggregates of amyloid in tissues [12, 20]. Having preserved the positive properties of Congo red we have synthesized its fluorine-based analogue, DSNAF. This analogue, as opposed to Congo red, lacks conformation mobility across the links between the benzene rings. The reduction of the number of possible conformation states of the DSNAF molecule and its planar structure allow for a significant increase of fluorescence of complexes of this dye with amyloidogenic protein fibrils. Thus, in the preliminary *in vitro* studies [21], it has been shown by us that during formation of complexes of this compound with the fibrils of lysozyme, beta2-microglobulin, and insulin, substantial growth of the dye fluorescence intensification occurs in comparison with fluorescence in the presence of the monomers of these proteins [21]. On the basis of structural similarity of DSNAF and Congo red, the supposition has been made on the possibility of applying the developed dye for identification of amyloid in the human tissues.

Within the frames of the present investigation, it has been proved for the first time that DSNAF can be successfully used for the detection of amyloid in the human myocardium. The results obtained demonstrate that in case of using light microscopy, DSNAF is quite comparable with Congo red by the intensity of amyloid staining. Absence of differences in the quantity and distribution of amyloid deposits during the analysis of the series sections speaks of the fact that specificity of DSNAF is similar to that of Congo red. Some differences in the color of amyloid fibrils have been noted: when binding to Congo red the deposits become pink colored, whereas binding to DSNAF they acquire a crimson hue. It is likely to be due to the differences in the spectra of absorption and emission of these stains (in case of DSNAF, the peaks of absorption and emission are shifted to a long-wave region of the spectrum).

Interestingly, in contrast to the amyloid aggregates stained with Congo red which are green in the polarized light due to the phenomenon of birefringence [22], amyloid stained with DSNAF does not possess this property. This disadvantage is partly compensated by its ability to fluoresce in the red spectrum range. Confocal microscopy data showed that fluorescence intensity of DSNAF bound to amyloid fibrils in human myocardium is more than twice higher than that of Congo red. Besides, intensive background staining of the muscular tissue is observed in case of Congo red application. It is less evident in case of DSNAF and can be eliminated by using the dye solution of lower concentration (0.034%).

Increase of fluorescence intensity of the empty background (in those places where there is no tissue on the preparation) has been noted in the region of the fluorescence peak of Congo red and DSNAF. It may be due to the partial transition of the dye from the tissue to the embedding medium which, like the dye itself, is water-based. In this case, fluorescence of the dye not bound to amyloid fibrils is observed. It is important that this fluorescence is very low.

## Conclusion

A new fluorene-based analogue of Congo red, DSNAF, can be successfully used to identify amyloid deposits in histological sections of human myocardium. In terms of the specificity and intensity of amyloid staining, DSNAF is comparable to Congo red. The fluorescence intensity of DSNAF when binding to amyloid fibrils is significantly higher than the intensity of Congo red fluorescence (with a lower intensity of background fluorescence of heart muscle tissue). This is especially useful for identifying small deposits of amyloid in the human tissues which is important when using small biopsies. Our technology of amyloid detection based on the application of the Congo red analogue may be considered as a novel promising method of identifying amyloid aggregations in human tissues.

## References

[1] Schavlovsky M.M (2010). Ethiology and pathogenesis of amyloidoses: the molecular and genetic basis.. Meditsinskiy akademicheskiy zhurnal.

[2] Sipe J.D., Benson M.D., Buxbaum J.N., Ikeda S.I., Merlini G., Saraiva M.J., Westermark P (2016). Amyloid fibril proteins and amyloidosis: chemical identification and clinical classification International Society of Amyloidosis 2016 Nomenclature Guidelines.. Amyloid.

[3] Hassan W., Al-Sergani H., Mourad W., Tabbaa R (2005). Amyloid heart disease. New frontiers and insights in pathophysiology, diagnosis, and management.. Tex Heart Inst.

[4] Morais G.C.P., Arruda M.M., Bonadia J.C.A., Pozzan G (2014). Cardiac amyloidosis: a challenging diagnosis.. Autops Case Rep.

[5] Zhdanova E.A., Rameev V.V., Moiseev S.V., Kozlovskaya L.V., Safarova A.F. (2011). Cardiac amyloidosis.. Klinicheskaya farmakologiya i terapiya.

[6] Ukholkina G.B., Kuchin G.A., Bychkova O.P., Chikhirev O.A (2016). Cardiac amyloidosis in clinical practice.. Zhurnal serdechnaya nedostatochnost’.

[7] Ovcharenko S.I., Son E.A., Okisheva E.A., Sedov V.P., Makolkin V.I. (2007). Cardiac amyloidosis.. Klinitsist.

[8] Nonka T.G., Repin A.N (2015). Cardiac amyloidosis. Diagnostics and treatment of amyloid cardiomyopathy. Case report.. Kliniceskaa medicina.

[9] Linke R.P (2006). Congo red staining of amyloid: improvements and practical guide for a more precise diagnosis of amyloid and the different amyloidoses.. Protein misfolding, aggregation, and conformational diseases..

[10] Korzhevskii D.E., Gilyarov A.V. (2010). Osnovy gistologicheskoy tekhniki.

[11] Picken M.M (2010). Amyloidosis — where are we now and where are we heading?. Arch Pathol Lab Med.

[12] Clement C.G., Truong L.D (2014). An evaluation of Congo red fluorescence for the diagnosis of amyloidosis.. Hum Pathol.

[13] Sapozhnikov S.P., Karyshev P.B., Sheptukhina A.I., Nikolayeva O.V., Avruyskaya A.A., Mitrasov Y.N., Kozlov V.A (2017). Novel fluorescent probes for amyloid detection.. Sovremennye tehnologii v medicine.

[14] Gusel’nikova V.V., Gudkova A.Ya., Semernin E.N., Grudinin N.A., Krutikov A.N., Shavloskii M.M., Mil’man B.L., Korzhevskii D.E., Mikhailova E.V., Kaminskaya E.V., Mikhailov V.M. (2017). Characterization of amyloid deposits found in internal organs of MDX mice.. Cell Tiss Biol.

[15] Gusel’nikova V., Antimonova O., Fedorova E., Shavlovsky M., Krutikov A., Mikhailova E., Gudkova A., Mikhailov V., Korzhevskii D. (2018). Fluorescent characterization of amyloid deposits in the kidneys of mdx mice.. Eur J Histochem.

[16] Gusel’nikova V.V., Antimonova O.I., Fedorova E.A., Shavlovskii M.M., Krutikov A.N., Mikhailova E.V., Kaminskaya E.V., Gudkova A.Ya., Korzhevskii D.E., Mikhailov V.M. (2018). Fluorene derivative disodium salt as a new fluorescent dye for identification of amyloid deposits in the myocardium of mdx mice.. Cell Tiss Biol.

[17] Grigorev I.P., Korzhevskii D.E (2018). Current technologies for fixation of biological material for immunohistochemical analysis (review).. Sovremennye tehnologii v medicine.

[18] Antimonova O.I., Korzhevskii D.E., Shavlovsky M.M. (2018). Method of fluorescent identification of amyloid..

[19] Korzhevskii D.E., Sukhorukova E.G. (2013). Gistokhimicheskie metody okrashivaniya gistologicheskikh preparatov. V kn.. Morfologicheskaya diagnostika: podgotovka materiala dlya morfologicheskogo issledovaniya i elektronnoy mikroskopii.

[20] Cohen A.S., Calkins E., Levene C.I (1959). Studies on experimental amyloidosis. I. Analysis of histology and staining reactions of casein-induced amyloidosis in the rabbit.. Am J Pathol.

[21] Antimonova O.I., Grudinina N.A., Egorova V.V., Ilyina V.V., Zabrodskay Y.A., Ramsay E.S., Shabalin K.A., Protasov A.V., Yakimov A.P., Polukeev V.A., Shavlovsky M.M (2020). Time machine: can a dye from 1928 be re-purposed for modern, fluorescence-based detection of amyloid-like fibrils?. Dyes and Pigments.

[22] Dapson R.W (2018). Amyloid from a histochemical perspective. A review of the structure, properties and types of amyloid, and a proposed staining mechanism for Congo red staining.. Biotech Histochem.

